# The Effect of Advanced Motherhood on Newborn Offspring's Hippocampal Neural Stem Cell Proliferation

**DOI:** 10.1155/2016/6171352

**Published:** 2016-09-05

**Authors:** Bo Li, Ping Duan, Xuefei Han, Wenhai Yan, Ying Xing

**Affiliations:** ^1^Department of Physiology, Zhengzhou University, Zhengzhou, Henan 450001, China; ^2^Stem Cell Research Center of Medical College, Zhengzhou University, Zhengzhou, Henan 450052, China; ^3^Department of Pathophysiology, Zhengzhou University, Zhengzhou, Henan 450001, China

## Abstract

*Objective*. To investigate the effect of advanced motherhood on rat hippocampal neural stem cell proliferation.* Methods*. Female parents were subdivided into control and old mother group by age, and neural stem cells were cultured from hippocampal tissues for 24 h newborn offspring. The diameter and numbers of neurospheres were examined by microscopy, and differences in proliferation were examined by EdU immunofluorescence, CCK-8 assay, and cell cycle analysis.* Results*. The number of neurospheres in the old mother group after culture was lower than the control group. Additionally, neurospheres' diameter was smaller than that of the control group (*P* < 0.05). The EdU positive rate of the old mother group was lower than that of the control group (*P* < 0.05). CCK-8 assay results showed that the absorbance values for the old mother group were lower than that of the control group at 48 h and 72 h (*P* < 0.05). The proportions of cells in the S and G2/M phases of the cell cycle for the older mother group were less than that found for the control group (*P* < 0.05).* Conclusion*. The proliferation rates of hippocampal NSCs seen in the older mother group were lower than that seen in the control group.

## 1. Introduction

 Delayed child-bearing has become increasingly common in human reproduction due to increasing trends in economic and social pressures in today's world. Moreover, the delayed child-bearing age might affect mental development of the offspring—this possibility has attracted increasing attention recently [1]. An age greater than 30 at the time of motherhood is known to affect the quality of the offspring [2], increase the incidence of hypospadias [3], increasing the likelihood of autism spectrum disorders [4, 5], and affecting the intellectual disability [6].

In mouse models, studies have found that offspring of older mothers showed a higher mortality rate before the weaning period; in addition, not only was low birth weight seen, but also spontaneous activity and learning deficits are seen during adolescence [7]. However, other studies have shown quite the opposite effects and have demonstrated that the mother's age at pregnancy had no effect on intelligence evaluation of the newborn baby [8, 9]. Moreover, even in children of older pregnant mothers, higher intelligence test scores (IQ) were seen [10]. Therefore, whether child intelligence if adversely affected by onset of motherhood age remains in dispute.

The ability of learning and memory can reflect differences in intelligence, which is associated with new neuronal generation in the hippocampus [11, 12]. Delayed motherhood was found to lead to unique patterns of rat hippocampal gene expression in offspring as compared to the reference group [13]. Neural stem cells are highly localized to the hippocampal dentate gyrus. Studies showed that, with increased neural stem cell division and proliferation, the stronger the ability to learn and develop effective memory [14, 15]. To date, the effect of delayed motherhood on hippocampal neural stem cell proliferation has not been previously reported.

By determining proliferative characteristics of the offspring between younger mothers and older mothers, the aim of this study was to examine differences at the cellular level.

## 2. Materials and Methods

### 2.1. Materials

#### 2.1.1. Animals

Newborn 24-hour-old Sprague-Dawley male rats (Animal Center of Zhengzhou University, Zhengzhou, China) were used in these experiments. Virgin females were subdivided into two groups by age: (1) 3 months old (the control group, *n* = 6) and (2) 12 months old (the old mother group, *n* = 6); females in the two groups were housed with randomly selected 3-month-old male rats.

All rat experiments were conducted in terms of the “Guidance on the Care and Use of Laboratory Animals” (2006, People's Republic of China National Science and Technology Committee Notice). Animal studies were approved by the Animal Ethics Committee of Zhengzhou University.

#### 2.1.2. Reagents

The reagents used in this study were 0.25% Trypsin-EDTA (1x); DMEM culture medium; fetal bovine serum (FBS); trypsin-EDTA and B27 (Gibco, USA); basic fibroblast growth factor (bFGF); epidermal growth factor (EGF) (PeproTech, London, UK); phycoerythrin- (PE-) conjugated anti-nestin antibody, which is a neural progenitor specific marker; propidium iodide (PI)/RNase staining buffer; Cytofix/Cytoperm kit (BD biosciences, Franklin Lakes, NJ, USA); 4,6-diamidino-2-phenylindole (DAPI) Nuclear Labeling Kit (GenView, Houston, TX, USA); cell proliferation assay (CCK-8 kit; KeyGEN BioTECH Corp., Ltd. Shanghai, China); and 5-ethynyl-2′-deoxyuridine (EdU) in vitro DNA proliferation assay kit (Rui Bo Guangzhou Biotechnology Limited Company, China).

### 2.2. Isolation, Purification, and Culture of NSCs

Neurospheres were generated from isolated NPCs in the hippocampal area of postnatal 24-hour-old rat. Briefly, rat brains were coronally sectioned, and the hippocampal area was obtained, which was followed by tissue and cellular dissociation. Isolated cells were cultured at a density of 5 × 10^5^ cells/mL in DMEM-F12 proliferation medium containing 2% B27 supplement, 20 ng/mL bFGF, and 20 ng/mL EGF. The cultures were observed and photographed daily under a phase contrast microscope (Model CKX41, Olympus, Japan). After 2 passages, cells were cultured for 72 and 96 h, Olympus imaging analysis system was employed to observe of neurospheres, and the number and diameter of neurospheres were counted and measured separately in 10 randomly selected microscopic fields (100x) for each flask.

### 2.3. Immunocytochemistry

Neurospheres were seeded onto coverslips that were precoated with poly-L-lysine (0.01 mg/mL, Sigma-Aldrich, St. Louis, MO, USA) for 5 h until the neurospheres had adhered, following which they were washed in PBS three times and fixed in 4% paraformaldehyde (PFA) for 30 min. Fixed cells were blocked in 0.3% Triton X-100 supplemented with PBS and 5% goat serum for 1 h at room temperature. Slides were washed in PBS three times and incubated with PE-conjugated nestin antibody (1 : 100) for 2 h at 37°C in the dark. Nuclei were counterstained with DAPI (1 : 10000) for 10 min at room temperature. Epifluorescence observation and photodocumentation were done using an Olympus BX51 microscope (Olympus, Japan) that was equipped with a Spot*™* digital camera (Diagnostic Instrument Inc., USA).

### 2.4. Flow Cytometry

Neurospheres were dissociated into single cells and resuspended in PBS. After centrifugation (179 ×g, 5 min, room temperature), cells were treated in accordance with the instructions that accompanied the BD Cytofix/Cytoperm assay kit. In this assay cells were incubated with 500 *μ*L of fixation/permeabilization solution that were then incubated for 20 min in the dark at room temperature. After centrifugation, cell pellets were incubated with 2 mL of BD perm/wash buffer for 10 min and then incubated with PE-conjugated-nestin antibodies (1 : 20) for 1 h at 4°C in the dark. Cells were then washed and resuspended in 300 *μ*L of PBS containing 1% paraformaldehyde. The ratio of nestin positive cells were analyzed by flow cytometry (BD FACSCanto II) with Data-Interpolating Vibrational Analysis (DIVA) software v. 5.0.

### 2.5. EdU Staining

Neurospheres were dissociated into single cells by Accutase and resuspended in PBS. The cells were seeded at a density of 1 × 10^5^ cells/mL into 24-well culture plates. EdU staining was conducted using a Cell-Light*™* EdU staining assay kit, according to the manufacturer's protocol. Cells were incubated with EdU (1 : 5000) for 24 h and then resuspended with DMEM-F12 proliferation medium and seeded onto coverslips that were precoated with poly-L-lysine and then incubated for 6 h in 37°C, 5% CO_2_. The culture supernatant was then removed, and 2 mg/mL glycine solution was added for 10 min at room temperature. Next, cells were rinsed in PBS for 5 min and the sections were permeabilized with 0.5% Triton X-100 in PBS for 10 min and washed twice with PBS for 10 min per wash. Cells were incubated with Apollo staining reaction solution for 30 min in the dark. Cells were washed twice in PBS that contained 0.5% Triton X-100 for 10 min per wash. Next, cells were counterstained with Hoechst 33342 for 30 min in the dark to stain the nuclei. The slides were then washed twice with PBS for 3 min per wash and observed immediately by fluorescence microscopy at a magnification of ×400.

### 2.6. CCK-8 Assay

Neurospheres were dissociated into single cells by Accutase and resuspended in PBS. The cells were seeded at a density of 1 × 10^5^ cells/mL in nine culture plates. The growth rates of the cells were then determined by CCK-8 assay. Next, cells were incubated in 10 *μ*L CCK-8 working solution at 0, 24, 48, and 72 h, followed by incubation for 2 h at 37°C. The absorbance was finally measured at 450 nm using a model 3550 microplate reader (Bio-Rad Laboratories, Inc., Hercules, CA, USA).

### 2.7. Cell Cycle Analysis by Flow Cytometry

Neurospheres were dissociated into single cells by Accutase and resuspended in PBS. Cells were then washed three times in PBS and then resuspended in 70% chilled ethanol and fixed at −20°C overnight. The cells were then washed and resuspended in 500 *μ*L PI/RNase Staining Buffer and incubated at room temperature for 30 min. Cell cycle analysis was performed by flow cytometry. Data from 30,000 cells were collected for each data file (Figure 4).

### 2.8. Statistical Analysis

Data are described as mean ± standard deviation. Statistical analysis of the data was carried out by Student's* t*-test using the SPSS v. 13.0 software program (SPSS, Inc., Chicago, IL, USA). An alpha value of *P* < 0.05 was considered a statistically significant difference.

## 3. Results

### 3.1. NSC Culture and Detection

**Figure 1:**
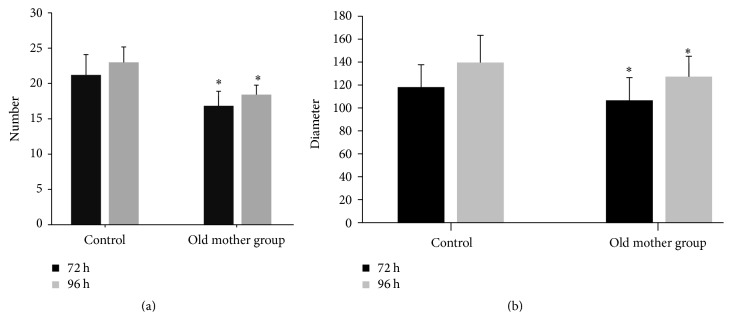
The number and diameter of the old mother group after NSCs were cultured for 72 and 96 h. ^*∗*^ *P* < 0.05 versus the control group (a, b). *n* = 6.

Neurospheres could be seen after hippocampal neural stem cell primary culture for three days, and the third generation of the cultured cells were observed. The number of neurospheres in the old mother group at 72 h and 96 h were lower than that seen in the control group, that is, 16.90 ± 2.08 versus 21.30 ± 2.91 and 18.50 ± 1.35 versus 23.10 ± 2.18, respectively (Figure 1(a)). The diameters of the neurospheres were also recorded in the old mother group and were 109.16 ± 20.22 mm at 72 h and 130.24 ± 18.75 at 96 h as compared to that seen in the control group, which were 121.02 ± 19.91 mm and 142.82 ± 24.26, respectively (*P* < 0.05) (Figure 1(b)).

### 3.2. Hippocampal NSC Identification and Detection of Nestin Expression

**Figure 2:**
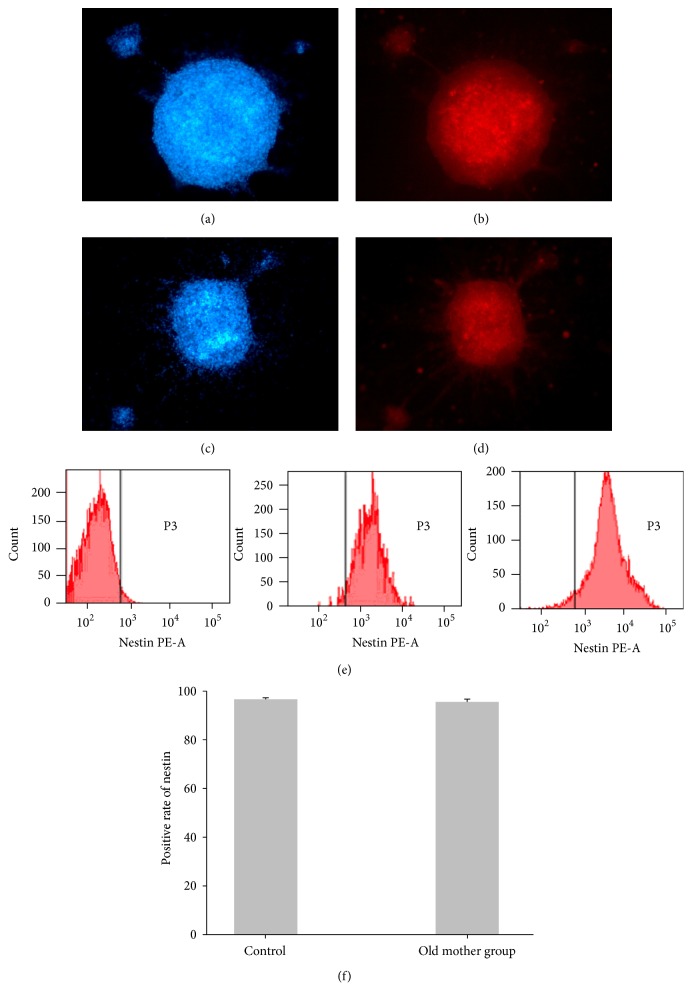
NSCs of both groups express the nestin protein (100x magnification). After NSCs were adhered for 5 h, DAPI and PE-conjugated nestin were costained, and fluorescence values were observed by microscopy (control group (a) and (b) and the older mother group (c) and (d)). Flow cytometry analysis in the control group (e, middle) and old mother group (e, right), respectively. Values are expressed as mean ± standard deviation. *n* = 6.

Results from immunocytochemistry demonstrated that the nestin protein were expressed in both groups. Flow cytometric analysis showed that nestin positive rates were 95.2% ± 1.18% in the old mother group as compared to 96.3% ± 0.60% in the control group, an observation that was not statistically significant (Figure 2).

### 3.3. Detection of NSCs Proliferation by EdU Labeling and CCK-8

**Figure 3:**
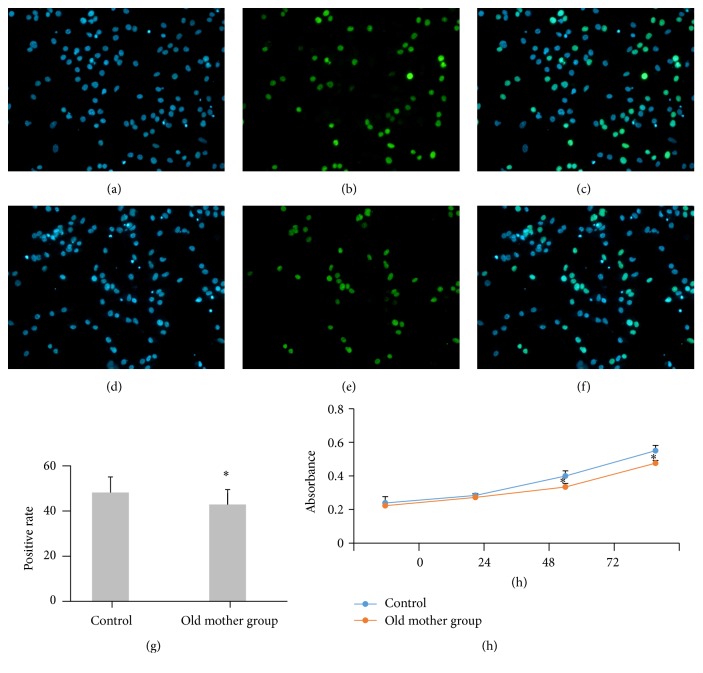
Proliferation detection of two groups. EdU stains were used to analyze the proliferation of NSCs, which showed (control group (a), (b), and (c) and old mother group (d), (e), and (f); 200x magnification) that cells were displaying a blue fluorescence after Hoechst 33342 staining (a and d) and green fluorescence after Apollo staining (b and e). The combined image (c and f) revealed that the EdU positive rate of the old mother group was lower than the control group. ^*∗*^ *P* < 0.05 versus the control group (g). CCK-8 assay revealed that the absorbance values of the old mother group was lower than that of the control group at 48 h and 72 h. ^*∗*^ *P* < 0.05 versus the control group (h). *n* = 6.

**Figure 4:**
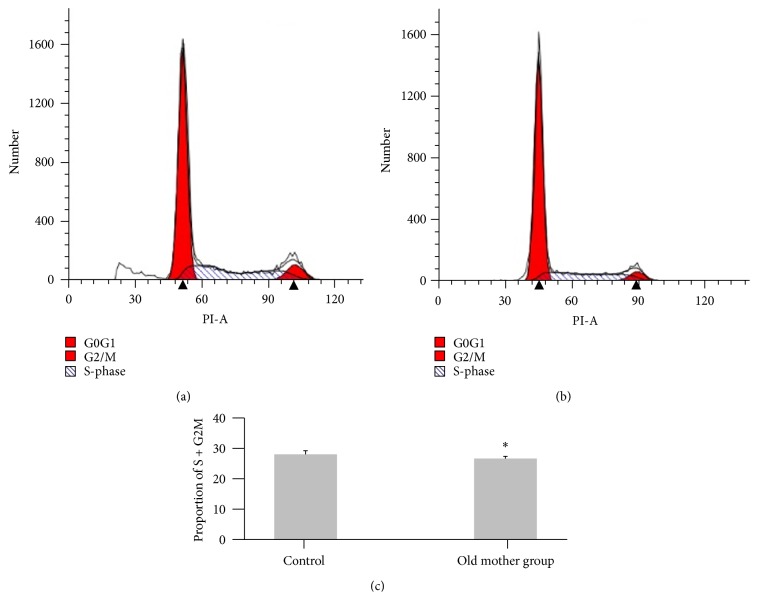
Cell cycle analysis of both groups. Cell cycle analysis of NSCs was detected by flow cytometry (a and b). ^*∗*^ *P* < 0.05 versus the control group (c). *n* = 6.

EdU positive rate of the old mother group was 43.32% ± 5.13% as compared to that of the control group of 48.58% ± 6.99% (*P* < 0.05) (Figure 3). Rat hippocampal NSC growth curves were drawn according to cell absorbance values, as shown in the figure. The absorbance values had increased from 0 h to 96 h in each group and the differences were statistically significant. The absorbance value of the old mother group was lower than that seen in the control group at 48 h and 72 h (*P* < 0.05).

### 3.4. Flow Cytometric Detection of Cell Cycle

To determine whether the proliferation of both groups was different, flow cytometry analysis was used based on determining the DNA content in nuclei that had been stained by incubation with PI. The proportions of cells in the S + G2/M phases of the cell cycle for the old mother group was 26.8 ± 0.7, whereas that for the control group cells was 28.2 ± 1.2% (*P* < 0.05). These results indicated that the proportion of cells in the S phase of the cell cycle was significantly increased, which was accompanied by a decrease in proportion of cells in the G0/G1 phase, when compared with nontreated cells. This suggested that rat hippocampal NSC proliferation may have displayed differences due to the age of the mother.

## 4. Discussion

Studies have previously shown that a newborn's growth and intellectual development might be affected by heredity and environment factors [16], such as the health of the parents, and such diverse factors as marriage between first cousins [17]. In addition, the mother's age at conception and how this influences a child's growth and general intelligence has increasingly concerned researchers in the field in recent years. Recent studies of a large sample survey of Swedish men, aged 17–20 years old, tested the intelligence scores of the youth and used their siblings to contrast the observations and reduced or eliminated factors other than child-bearing age. The results confirmed that the father's age did not influence the child's IQ and that the mother' age above 30 years of age adversely affected the child's IQ [18].

Learning and memory are the main measurements of human mental development. Moreover, electrical activity and biochemical metabolism in hippocampal neurons are closely related to higher brain function [19, 20]. At present, the hippocampus is thought to be the key focus area of learning and memory.

Neural stem cells (NSCs) are self-renewing, multipotent cells that generate the main phenotype of the central nervous system. NSCs also undergo asymmetric cell division into two daughter cells, wherein one is specialized and the other is a nonspecialized subtype [21, 22]. NSCs primarily differentiate into astrocytes, and oligodendrocytes, and neurons and show the corresponding cell phenotype, morphological structure, and biological features [23, 24]. Studies have previously shown that the proliferation capacity of neural stem cells in hippocampal tissues is associated with learning and memory ability [25, 26]. Thus, the division and proliferation of neural stem cells may play an important role in learning and memory in the brain.

Thus, in order to explore whether motherhood age influences a child's mental development, our study attempted to demonstrate that neural stem cell proliferation was the breakthrough point. This was done in an experimental design whereby the rat offspring's hippocampal NSCs from young and older mothers were cultured and contrasted. We found that both groups highly expressed the nestin protein, but there were no significant differences between them. The growth status of both groups were observed under the microscope and showed that the diameter of the neurospheres of the old mother group was much higher than that of the control group after culture for 72 h and 96 h. In addition, the number of neurospheres of the old mother group was higher than that of the control group—an observation suggesting that the proliferative ability of rat NSCs in the old mother group was lower.

To further explore the difference in the capacity of NSCs to proliferate, both groups were stained with EdU and assessed by the CCK-8 assay to identify and measure cellular proliferation. Observations found that the proliferation cell ratio of young rat hippocampal NSCs was higher than that found in older hippocampal NSCs. Moreover, cell cycle detection by flow cytometry verified the results. Thus, we concluded that, for the old mother group, nestin expression of hippocampal NSCs was not different from that seen in the control group. However, the proliferative activity was lower than that seen in the control group.
